# Ecophysiology of an Avian Invader: Body Condition and Metabolic Rate Adjustments to Ambient Temperature

**DOI:** 10.1111/1749-4877.12969

**Published:** 2025-03-26

**Authors:** Marina Sentís, Cesare Pacioni, Emma Bossuyt, Andrey Bushuev, Anvar Kerimov, Luís Reino, Luc Lens, Diederik Strubbe

**Affiliations:** ^1^ Centre for Research on Ecology, Cognition and Behaviour of Birds (ECoBird) Ghent University Ghent Belgium; ^2^ Laboratory of Molecular Entomology and Honeybee Pathology, Department of Biochemistry and Microbiology, Faculty of Sciences Ghent University Ghent Belgium; ^3^ Department of Vertebrate Zoology, Faculty of Biology M.V. Lomonosov Moscow State University Moscow Russia; ^4^ CIBIO, Centro de Investigação em Biodiversidade e Recursos Genéticos, InBIO Laboratório Associado, Campus de Vairão Universidade do Porto Vairão Portugal; ^5^ BIOPOLIS Program in Genomics, Biodiversity and Land Planning, CIBIO Campus de Vairão Vairão Portugal; ^6^ CIBIO, Centro de Investigação em Biodiversidade e Recursos Genéticos, InBIO Laboratório Associado, Instituto Superior de Agronomia Universidade de Lisboa Lisboa Portugal; ^7^ Research Institute for Nature and Forest (INBO) Herman Teirlinckgebouw Brussel Belgium

## Abstract

This study examines the ecophysiological responses of common waxbills to temperature variation in Portugal. We measured body condition and basal metabolic rate (BMR) during summer and winter across two regions in Portugal. Body condition was negatively correlated with temperature, while the relationship between BMR and temperature varied seasonally. In summer, BMR decreased with increasing temperature, but in winter, it remained low and stable, indicating physiological adjustments to seasonal changes.

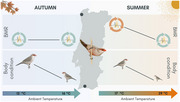

## 1 Introduction

Predicting how species are likely to respond to changes in their environment brought about by human activities is crucial for conservation planning aimed at stemming the current biodiversity crisis (Guisan et al. 2013; Schwartz 2012). To persist, animals must maintain a balance between energy acquisition and energy expenditure, and this challenge is particularly pronounced for endotherms (Kronfeld‐Schor and Dayan 2013). Organisms can respond to changing conditions through a range of physiological, morphological, and behavioral adjustments (Agrawal 2001), and body mass and metabolic rates are among the most important factors influencing the thermoregulatory capacity of animals (Barnett and Briskie 2011). For example, in colder, seasonal environments, individuals often adjust their body mass in response to lower temperatures, for example, by accumulating fat reserves (Gosler 2002; Polo et al. 2007), and elevating their metabolic rates to meet the increased thermoregulatory demands of cold winters (Liknes et al. 2002; McKechnie et al. 2015).

Metabolism is most commonly quantified using the basal metabolic rate (BMR)—the minimum rate of energy expenditure, measured within the thermoneutral zone of a species, required by a resting, post‐absorptive individual to maintain normothermic body temperature (McNab 2012). BMR typically represents a significant portion of an animal's energy budget and is considered to reflect physiological maintenance costs (Broggi et al. 2022; McKechnie 2008). The causes and consequences of genetic variation and phenotypic plasticity in BMR have attracted considerable research attention (Burton et al. 2011; Nafstad et al. 2023; Pacioni et al. 2024). Metabolic rates are generally considered to be repeatable and heritable (Bushuev et al. 2011; Nilsson et al. 2009), but they are also highly plastic, for example, in response to season, temperature, habitat, and behavior (McNab 2012). Several studies have documented a correlation between BMR and thermogenic capacity—which is typically quantified as an animal's summit metabolism (Liknes et al. 2002; Liknes and Swanson 1996). Such a relationship is thought to be indirect, as both BMR and summit metabolism may respond in parallel but independently to environmental pressures such as cold (Petit et al. 2013; Swanson et al. 2023; Swanson et al. 2012; Vézina et al. 2006). Correlations then arise, for example, when colder temperatures may select for more muscle mass to increase shivering thermogenesis capacity, with a correlated increase in BMR to support increased food processing, with the growth of central organs, such as the gut, the liver, the kidney, the heart, and the lungs (Chappell et al. 1999; Mckechnie and Swanson 2010; Swanson 2010; Swanson et al. 2020).

How species cope with the energetic challenges posed by the environment is of direct relevance to predicting the success of invasive alien species. Although invasion success is generally greater when species are introduced into climates similar to those of their native range (Blackburn and Duncan 2001; Redding et al. 2019), niche expansion into novel climates is not uncommon (Hayes and Barry 2008; Liu et al. 2022; Mahoney et al. 2015; Shwartz et al. 2009). In novel environments, invaders can experience rapid changes in phenotypic traits (Tingley et al. 2012; Whitney and Gabler 2008) through plasticity or trait evolution over time (Agrawal 2001; Prentis et al. 2008). For example, Louppe et al. (2018) found that differences in metabolic rates between core and expanding edge populations of invasive African clawed frogs (*Xenopus laevis*) in France led to different phenotypes, with animals from the periphery being able to invest more of their energy in locomotor performance, and thus to dispersal. However, there is a paucity of empirical studies examining the effects of environmental conditions on ecophysiological traits in invasive alien populations (Briscoe et al. 2023), especially for endothermic vertebrate invaders. Understanding the mechanisms that govern how animals adjust their mass and metabolic rates can help predict how introduced species are likely to respond to novel climates, improving predictions of likely geographic distributions in their new ranges (Strubbe et al. 2023) and aiding the development of effective management strategies (McCann et al. 2018).

Here, we investigate body condition and BMR regulation in invasive common waxbills (*Estrilda astrild*). Common waxbills are small sub‐Saharan estrildid finches (7–9 g) that have been introduced worldwide as a result of the pet trade (Cardoso and Reino 2018; Stiels et al. 2011) and they have been particularly successful in parts of the Iberian Peninsula (Reino and Silva 1998; Reino 2005). The native range of common waxbills extends from Ethiopia to South Africa and includes 16 subspecies (del Hoyo et al. 2010). Genotyping studies suggest that Portuguese populations have a mixed ancestry (Cardoso et al. 2014) and likely originated from former Portuguese colonies like Mozambique and Angola (Cardoso and Reino 2018). Since their introduction in central Portugal in the early 1960s, invasive waxbills have greatly expanded their range, and populations in the northern Iberian Peninsula represent the northernmost distribution limit of the species. Across the Iberian Peninsula, invasive birds experience a colder and less predictable climate compared to most of their native range (Stiels et al. 2011), and their population numbers appear to fluctuate with winter severity (Franch et al. 2021). The geographic spread of common waxbills across the Iberian Peninsula is influenced by both climatic and habitat factors (Cardoso and Reino 2018; Santana et al. 2024), but studies investigating the ecophysiological basis of this invasion are largely lacking. Pacioni et al. (2023) studied a population of captive‐bred common waxbills kept in aviaries in Belgium and found that body mass remained constant from summer to autumn, but metabolic rate decreased. However, extrapolation of findings from captive‐bred birds with access to permanent shelter and ad libitum food to wild, free‐ranging individuals remains problematic (Geiser and Ferguson 2001).

In this study, we quantify BMR and body condition of common waxbills invasive across Portugal. Portugal's diverse geography, influenced by its position between the Atlantic and the Mediterranean seas and characterized by a varied relief, produces different regional climates. The south tends to have hot summers and mild winters, while the north has warm summers and generally cool, wet winters. To assess how the ecophysiological traits of waxbills vary with ambient temperature, we captured and measured common waxbills at two sites in Portugal, one in the north and another one in the south, in both summer and late autumn, which allowed us to capture significant climatic variation. We expect that in relation to the higher thermoregulatory costs imposed by colder ambient temperatures, waxbill body condition and BMR will be negatively related to ambient temperatures.

## 2 Materials and Methods

### 2.1 Study Sites and Captures

The fieldwork was conducted at two different sites in Portugal during late autumn (October 27–December 1, 2021) and summer (June 15–July 15, 2022), with sampling taking place over approximately 3 weeks per site during each season. The selection of these two sites was aimed at introducing variation in ambient temperatures. The northern site, located near Caminha (41.846, −8.801), is characterized by warm (but not hot) dry summers with mild to cold rainy winters (Csb) (Geiger 1954). The southern site, located near Estômbar (37.171, −8.485), is characterized by hot to very hot, dry summers and mild winters (Csa) (Geiger 1954) (Table S1). Although the northern and southern regions of Portugal differ in general landscape characteristics, birds at both sites were captured in reed beds of similar structure and age.

Common waxbills (*n* = 277) were captured using 12‐m mist nets with 16×16 mm mesh (Ecotone), mainly within reed beds, and lured with recordings of waxbill vocalizations. Birds were aged on the basis of bill color (black for juveniles and red for adults) and sexed based on their under‐tail coverts (black for males and brown for females) (Table S1). Body mass was measured to the nearest 0.1 g with a digital scale, along with other morphological measurements such as tarsus, wing, and tail lengths, which were measured to the nearest 0.1 mm using a digital caliper. Up to seven birds per day were then placed in cages (35 cm × 29 cm × 23 cm) with ad libitum access to food (finch mix and millet) and water. They were then transported to a nearby facility equipped with respirometry equipment for further measurements. Birds with brood patches (*n* = 19) were released immediately after the morphological measurements and were not included in the respirometry trials. All birds included in the respirometry measurements were released at their original capture locations the morning after the measurements. Ethical approval for this study was obtained from the Ethical Committee VIB/UGent—Faculty of Science, with reference number EC2021‐055. Local ringing permits were obtained from the Institute for Nature Conservation and Forests (ICNF) in Portugal (N° 256/2021, N° 257/2021, N° 258/2021).

### 2.2 Basal Metabolic Rate (BMR) and Body Condition

An open flow‐through respirometry system measuring O_2_ consumption (VO_2_; mL/min), CO_2_ production (VCO_2_; mL/min), and water vapor pressure (kPa) was used (Field Metabolic System, FMS‐3, Sable Systems). The FMS‐3 was calibrated regularly. The O_2_ sensor was calibrated every 2–3 days at 20.94% by using the fixed‐span mode with ambient air flowing through a Drierite column (Lighton 2018). The CO_2_ and the water‐vapor sensors were set to zero with pure nitrogen (N_2_) right before the measurement period. The CO_2_ sensor was also spanned before the measurements using a reference gas consisting of 99% N_2_ and 1% CO_2_.

To measure the BMR, individuals were fasted for 2 h prior to respirometry and then weighed to the nearest 0.1 g. Birds were placed in airtight plastic chambers (1.1 L) overnight. Two pumps supplied atmospheric air, which was divided into eight separate streams. Each stream was directed to a mass flow meter (FB‐8, Sable Systems), set to deliver 650 mL/min of air to each of the eight metabolic chambers—seven housing individual birds and one serving as an empty reference chamber for baseline measurements. The excurrent airstreams were connected to a multiplexer (RM‐8, Sable Systems), which allowed the airstream from one chamber to be sampled independently of the others. The excurrent air from both the bird and baseline channels was alternately subsampled and pulled through the FMS‐3, with a subsampling flow rate of 200 mL/min. During a measurement session (i.e., during the night, from dusk to dawn), birds were measured alternately in cycles, along several baselines. Within each cycle, each bird was measured for either 10 min (when at least four birds were captured that day and available for measurement) or 20 min (when fewer than four birds were available). The total duration of each session varied seasonally, ranging from 9.5 h in autumn to 7.5 h in summer due to shorter nights. On average, per night, 6.2 birds were measured in autumn and 4 birds in summer. All chambers were placed inside a dark insulating foam box that was equipped with heating wires and a small fan to maintain a temperature of 28°C, which was within their TNZ (Pacioni et al. 2023; Sentís et al. 2023). The software ExpeData (Sable Systems) was used to record the measurements and extract VO_2_ (mL/min) using eq. 9.7 from Lighton (2018). The average minimum 5 min oxygen consumption was selected to estimate BMR overnight (Broggi and Nilsson 2023). After the measurements, waxbills were weighed again to the nearest 0.1 g, were fed ad libitum, and released at their site of capture.

Body condition was quantified using the residuals of log‐transformed body mass regressed on log‐transformed tarsus length (Schulte‐Hostedde et al. 2005). To standardize measurements and account for body mass differences throughout the day, body mass was always measured in the morning after BMR measurements.

### 2.3 Ambient Temperature Data

Ambient temperatures from each study site and period were obtained using the ERA5 reanalysis (Hersbach et al. 2023), which provides hourly estimates of ambient temperatures at 2‐m height with a spatial resolution of 0.25°. ERA5 collects this information from a combination of sources, including weather stations and satellites. These data sources are integrated using advanced weather forecasting models to produce consistent and reliable temperature estimates across the globe. Temperatures from the 7 days prior to the BMR measurements were used for the analyses (Bushuev et al. 2021). The AIC criterion was then used to rank a set of linear models and determine which temperature variables (mean, maximum, minimum, and average daily temperature differences) had the most significant effect on BMR and body condition. Mean and maximum temperatures showed similar results for BMR, while mean and minimum temperatures did for body condition. As a result, the mean temperature was selected for subsequent analyses. Further details can be found in Text S1, Table S3, and Figure S2.

### 2.4 Data Analyses

All statistical analyses were performed in R software v. 4.3.2 (R Core Team 2023). Linear regression models were used to assess how body condition and mass‐independent BMR, obtained as the residuals from the regression of log‐transformed BMR on log‐transformed body mass, varied with ambient temperature. In addition to ambient temperature, independent variables included location (south vs. north), season (summer vs. autumn), sex and age, and the interactions between temperature and location, and temperature and season. Non‐significant interactions and terms were removed from the model in a stepwise backward procedure until a minimal model with only significant variables (*p* value ≤ 0.05) remained. When a two‐factor interaction was significant, separate analyses were performed with each main effect separately. Shapiro–Wilk tests were used to check the normality of the residual values. Additionally, we used chi‐square tests of independence to assess whether sex and age distributions differed between sites.

### 2.5 Data and Code Availability

The original data and associated script are publicly available at: https://doi.org/10.6084/m9.figshare.25002596.v2

## 3 Results

**FIGURE 1:**
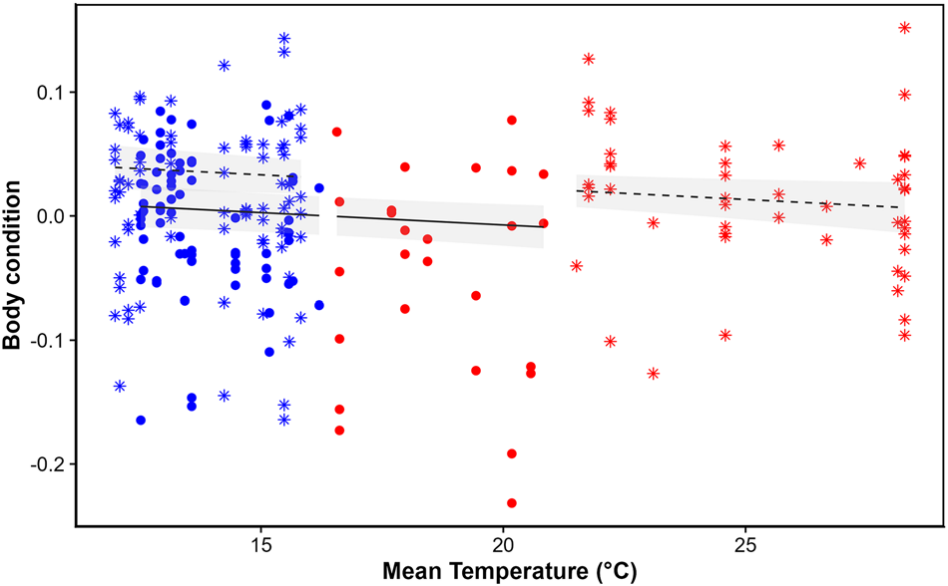
Body condition plotted against mean air temperature (°C) for the 7 days prior to BMR measurements obtained from ERA5. Blue = autumn, red = summer, dots and full line = northern Portugal, and stars and dashed line = southern Portugal.

**FIGURE 2:**
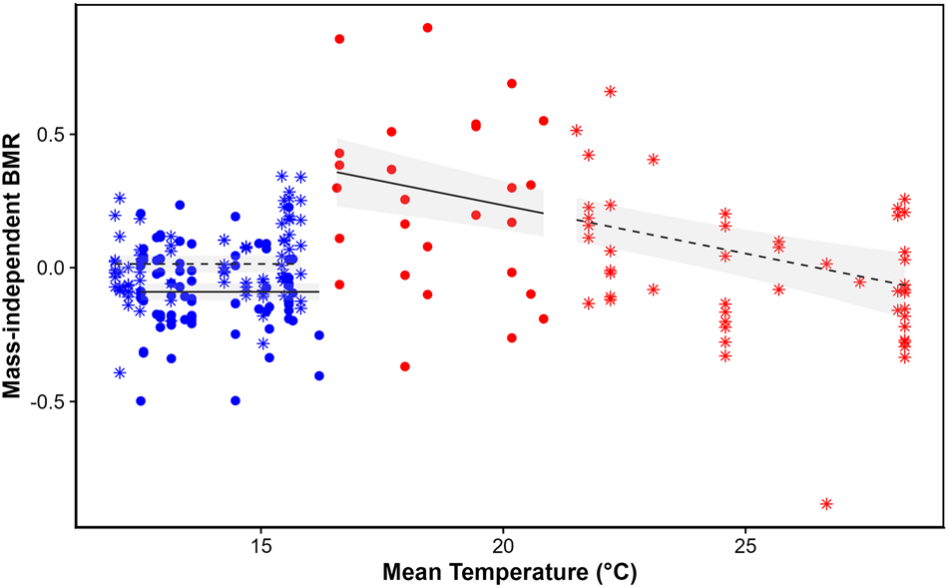
Mass‐independent BMR plotted against mean air temperature (°C) for the 7 days prior to BMR measurements obtained from ERA5. Blue = autumn, red = summer, dots and full line = northern Portugal, and stars and dashed line = southern Portugal.

Sex and age distributions did not differ between capture sites (*p* value = 0.65 and *p* = 0.96, respectively). Body condition was positively correlated with ambient temperature, regardless of season (model slope = −0.002 ± 0.0009, *p* = 0.024; Figure 1), was lower in juveniles compared to adults (−0.048 ± 0.0094, *p* < 0.0001) and was 4.1% higher in southern compared to northern Portugal (0.033 ± 0.0093, *p* < 0.001). (Log) body mass and (log) BMR were positively correlated (*r* = 0.33, *p* < 0.0001; see Figure S1 and Table S2). The relationship between mass‐independent BMR and ambient temperature differed between summer and autumn (interaction *p* < 0.0001, Figure 2; see Figure S3 for whole‐body BMR). In summer, BMR decreased with increasing ambient temperature (−0.037 ± 0.008, *p* < 0.0001), and during the coldest summer days, BMR was 48.37% higher than during the warmest days. In autumn, BMR was independent of ambient temperature (0.013 ± 0.0094, *p* = 0.18; Figure 2) and was higher in southern compared to northern Portugal (0.104 ± 0.023, *p* < 0.0001). BMR of northern waxbills thus changed with season and was lower in autumn than in summer (Figure 2).

## 4 Discussion

Our results show that common waxbills from the southern population generally have a higher body condition than northern birds and that as expected, in both areas, body condition is higher when temperatures are lower. BMR and temperature correlated negatively only in the summer months. In autumn, waxbill BMR was independent of temperature, and waxbills from the northern population decreased their BMR in autumn compared to summer. Thus, we show that waxbill body condition and metabolic rates vary with ambient temperature in a geographically dependent manner, potentially representing phenotypic responses contributing to the success of this species.

### 4.1 Influence of Temperature on Body Condition

Lower ambient temperatures place greater demands on the energetic requirements of birds (Cresswell 2009), and increasing fat reserves can reduce the risk of starvation (Davidson and Evans 1982). The upregulation of body reserves in response to decreasing temperatures is sometimes referred to as “winter fattening,” referring to the often observed increase in body mass from summer to winter in birds from mid to high latitudes (King and Farner 1966; Lehikoinen 1987). Much less is known about the strategies of birds from lower latitudes (Pravosudov and Grubb 1997). Merom et al. (2005) showed that the body mass and fat content of clamorous reed warbler (*Acrocephalus stentoreus*) in Israel varied as predicted by the winter fattening model. Similarly, in the Cape Town (South Africa) region, Lee et al. (2020) documented a ∼2% decrease in common waxbill body condition in response to climate change from 1988 to 2005, a period characterized by an annual maximum daytime warming rate of 0.035°C per year. Low‐latitude bird species generally have less variable body masses than non‐tropical species (Read et al. 2018). Our results however suggest that despite their subtropical origin, common waxbills may be able to increase their body condition, for example, to buffer themselves against potential food shortages. Although these changes remain modest in our study, they can still carry meaningful ecological implications for small birds (Van Buskirk et al. 2010; Jirinec et al. 2021). It should be noted that in our study, body condition was consistently higher in the southern region compared to the northern region, regardless of temperature and season. This suggests that factors beyond temperature may influence body condition, highlighting the need for further research.

### 4.2 Influence of Temperature on BMR Regulation

BMR is known to vary in response to a range of environmental factors, including ambient temperature. For example, Stager et al. (2016) conducted a comprehensive review of studies involving 40 bird species from diverse regions around the world and found that BMR correlated with the seasonality of temperature throughout the year. Bushuev et al. (2021) studied 134 free‐ranging tropical bird species in Vietnam and found a negative correlation between BMR and the difference between absolute maximum and minimum ambient temperatures within the week prior to capture and measurement. Numerous studies have also documented seasonal variation in the metabolism related to annual variation in temperature (McKechnie et al. 2015; Noakes et al. 2016), with breeding season BMR being higher, lower, or equal to winter BMR (McKechnie 2008; Swanson 2010). Here, we found that the relationship between waxbill BMR and ambient temperature varied between seasons. In summer, birds in both regions responded similarly, as indicated by the nearly identical slope of the relationship between BMR and ambient temperature in the north and south (Figure 2), despite no overlap in daily mean temperatures between the study sites (north Portugal: 16 to 21°C vs. 22 to 28°C for the south). In contrast, during late autumn, when temperatures in northern and southern Portugal were similar (12°C to 16°C), birds’ BMR did not vary with temperature and remained as low as the lowest recorded summer rate (Figure 2). Thus, we find that BMR remains relatively similar at the coldest and warmest extremes of the temperature range sampled.

Many avian species exhibit considerable phenotypic flexibility in their maintenance energy requirements, allowing them to adjust their BMR over relatively short time scales, ranging from days to weeks (Bushuev et al. 2021; McKechnie et al. 2007). The negative correlation between ambient summer temperature and BMR documented here can be attributed to several mechanisms that are not mutually exclusive. Higher temperatures may diminish the need for body heat generation (Anava et al. 2001), thereby conserving energy, which can then be redirected, for example, toward breeding activities (Salvante et al. 2010), which for Portuguese waxbills predominantly occur during late spring and early summer (Beltrão et al. 2021). Although the birds studied did not exhibit clear signs of breeding, such as brood patches, elevated stress hormones and increased thyroid activity are the potential internal mechanisms that could modulate bird energy expenditure (Vézina and Williams 2003; Wingfield and Farner 1993). Moreover, birds may also reduce their activity levels during peak temperature times, for example, to avoid overheating and/or minimize water loss rates, affecting BMR (Smit and McKechnie 2015; Speakman and Król 2010).

In contrast to the summer period, there was no relationship between waxbill BMR and ambient temperature during autumn. The relatively low BMR exhibited by wild invasive waxbills during the Portuguese autumn contrasts with our initial expectation of a higher metabolic rate in response to the colder temperatures. This finding is also in contrast to a recent study conducted by González‐Medina et al. (2023) on eight small‐bodied, Mediterranean‐resident native songbirds near the Portuguese border in Badajoz, southern Spain. They reported that these native birds increased their BMR by an average of about 9% in response to colder winter temperatures. Our results do align with those of Pacioni et al. (2023), who documented a decline in BMR from summer to winter in a captive waxbill population. In subtropical regions, several studies have suggested a reduction in winter BMR as an energy‐saving mechanism, contributing to improved winter survival (e.g., Bush et al. 2008; Smit and McKechnie 2010; Thabethe et al. 2013). Lovegrove (2000, 2003) suggested that while animals from high latitudes often show an increased BMR in winter, in the generally milder winters of the Afrotropical region, energy conservation becomes the primary seasonal metabolic response. Our findings may suggest that invasive waxbills mirror more closely the metabolic patterns observed for Afrotropical species than those of native European birds.

The observation that the autumn energy‐saving BMR approximates the values observed during the warmest summer days suggests that these values may represent the minimum BMR achievable for waxbills. However, further experimental studies with birds reared under standardized laboratory conditions and acclimated to different temperatures may be required to confirm this hypothesis. Furthermore, the reason why mainly waxbills from northern Portugal decreased their BMR in autumn, while birds from southern Portugal did not, remains unclear and may point to regional differences in environmental pressures. Additionally, with only two sites sampled over two seasons, we acknowledge that our ability to distinguish location effects from ambient temperature effects is constrained. Future research should therefore include multiple locations across different temperature gradients to better assess these effects. While our study benefits from a large sample size per site, stronger inferences could be made by distributing samples across such gradients.

## 5 Conclusions

In conclusion, this study reveals a complex interplay between ambient temperature, metabolic rate, and body condition in invasive common waxbills. Our findings indicate that waxbills have a higher body condition in colder environments, possibly in response to the increased thermoregulatory demands and potential food scarcities associated with these conditions. The observed seasonal variation in the relationship between BMR and temperature suggests that this species possesses the capacity for seasonal adjustments in metabolic function. However, the extent to which these metabolic changes are directly driven by thermoregulatory costs associated with climatic conditions, as opposed to other factors such as breeding cycles, habitat quality, or other non‐temperature‐related climatic factors, remains unclear from our data. Further research is necessary to determine whether and how variations in body mass and BMR contribute to enhanced survival and reproductive success. More research is also needed to investigate whether the observed fluctuations in body condition and BMR align with patterns seen within their native habitat, or if they constitute new phenotypic adjustments to the ecological challenges found in their non‐native range.

## Supporting information




**Table S1** Mean and standard deviation (SD) values for temperature (°C), mean precipitation (mm), total number of birds captured, and their sex (F: female; M: male) and age (A: adult; J: juvenile) for each location (NP: northern Portugal; SP: southern Portugal) and season. Temperature and precipitation data were obtained from the ERA5 dataset for the fieldwork period.
**Table S2** Results of a linear model describing the effect of body mass, location, season, sex and age on the whole‐body basal metabolic rate (BMR) of common waxbills
**Table S3** AIC scores for the effects of different temperature variables on body condition (body mass residuals) and mass‐independent BMR
**Figure S1** Relationship between (log)body mass (g) and (log)BMR (ml/min).
**Figure S2** Body condition (A, B) and mass‑independent BMR (C, D) plotted against 7‑day mean, minimum, and maximum air temperatures (°C) derived from ERA5.
**Figure S3** Whole‐body BMR (ml/min) plotted against mean air temperature (°C) for the 7 days prior to BMR measurements obtained from ERA5.
