# Supplementary material for: Ecophysiology of an Avian Invader: Body Condition and Metabolic Rate Adjustments to Ambient Temperature
Source: Integr Zool. 2025 Mar 26;21(3):602–8. doi: 10.1111/1749-4877.12969 (PMC13165713; doi:10.1111/1749-4877.12969)
Supplement: Supplementary file 1 — Table S1 Mean and standard deviation (SD) values for temperature (°C), mean precipitation (mm), total number of birds captured, and their sex (F: female; M: male) and age (A: adult; J: juvenile) for each location (NP: northern Portugal; SP: southern Portugal) and season. Temperature and precipitation data were obtained from the ERA5 dataset for the fieldwork period. Table S2 Results of a linear model describing the effect of body mass, location, season, sex and age on the whole‐body basal metabolic rate (BMR) of common waxbills Table S3 AIC scores for the effects of different temperature variables on body condition (body mass residuals) and mass‐independent BMR Figure S1 Relationship between (log)body mass (g) and (log)BMR (ml/min). Figure S2 Body condition (A, B) and mass‑independent BMR (C, D) plotted against 7‑day mean, minimum, and maximum air temperatures (°C) derived from ERA5. Figure S3 Whole‐body BMR (ml/min) plotted against mean air temperature (°C) for the 7 days prior to BMR measurements obtained from ERA5. [file INZ2-21-602-s001.pdf]

SUPPLEMENTARY MATERIALS

Table S1 Mean and standard deviation (SD) values for temperature (°C), mean precipitation (mm), total number of birds captured, and their sex (F: female; M: male) and age (A: adult; J: juvenile) for each location (NP: northern Portugal; SP: southern Portugal) and season. Temperature and precipitation data were obtained from the ERA5 dataset for the fieldwork period.

	**Autumn**		**Summer**	
	**NP**	**SP**	**NP**	**SP**
**Temperature (°C)**	13.4 ± 1.8	13.5 ± 2.1	17.5 ± 1.3	26.6 ± 2.9
**Precipitation (mm)**	74.54	8.29	31.71	0.03
**Total n of birds**	97	85	44	51
**Sex (F / M)***	41F / 51M	36F / 47M	21F / 22M	20F / 29M
**Age (A / J)***	43A / 25J	42A / 35J	31A / 13J	44A / 7J

*Sex and age could not be determined for some individuals

Table S2 Results of a linear model describing the effect of body mass, location, season, sex and age on the whole-body basal metabolic rate (BMR) of common waxbills

	**Estimate**	**SE**	**t-value**	**p-value**
Intercept	-3.25953	0.50949	-6.398	< 0.0001
Log(Body mass)	1.29891	0.24347	5.335	< 0.0001
Sex: Male (vs. Female)	0.04731	0.03257	1.453	0.1480
Age: Juvenile (vs. Adult)	0.08131	0.03640	2.234	0.0266
Location: SP (vs. NP)	-0.02139	0.03329	-0.642	0.5213
Season: Summer (vs. Autumn)	0.13768	0.03410	4.037	< 0.0001

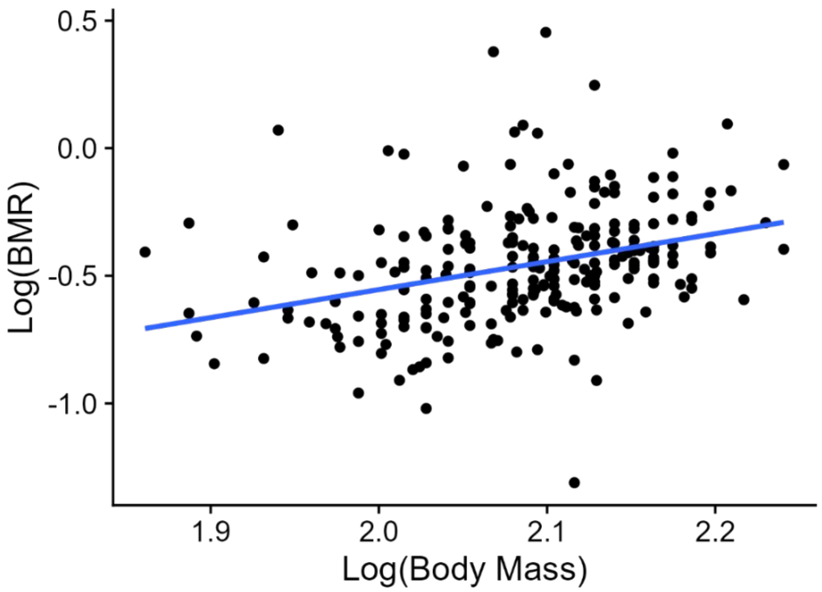

Figure S1 Relationship between (log)body mass (g) and (log)BMR (ml/min). Model slope = 1.096 ± 0.203, $R^2 = 0.110$, $p < 0.0001$.

SupMat Text 1. Ambient temperature selection

To obtain ambient temperatures, the ERA5 reanalysis was used (Hersbach et al., 2023), which provides hourly estimates for ambient temperatures at 2 m altitude at a 0.25° spatial resolution. For the analyses, the temperatures from the 7 days preceding the BMR measurements were used (Bushuev et al., 2021). The Akaike information criterion (AIC) criterion was then employed to rank a set of linear models and determine which temperature variables (mean, maximum, minimum, and average daily temperature differences) had the most significant impact on the BMR. For mass-independent BMR, minimum and range of temperature had high AIC values compared to mean and maximum temperatures (delta AIC > 10). Maximum and mean temperature had similar results (delta AIC ~ 4.2). For body condition, minimum and mean temperature had the best AIC scores (delta AIC ~ 2.4). Therefore, mean temperature was selected for all the analyses.

Table S3 AIC scores for the effects of different temperature variables on body condition (body mass residuals) and mass-independent BMR

	**Mean temperature (°C)**	**Maximum temperature (°C)**	**Minimum temperature (°C)**	**Temperature range (°C)**
**Body condition**	-551.2	-548.8	-553.6	-548.3
**Mass-independent BMR**	-97	-101.2	-86.1	-86.0

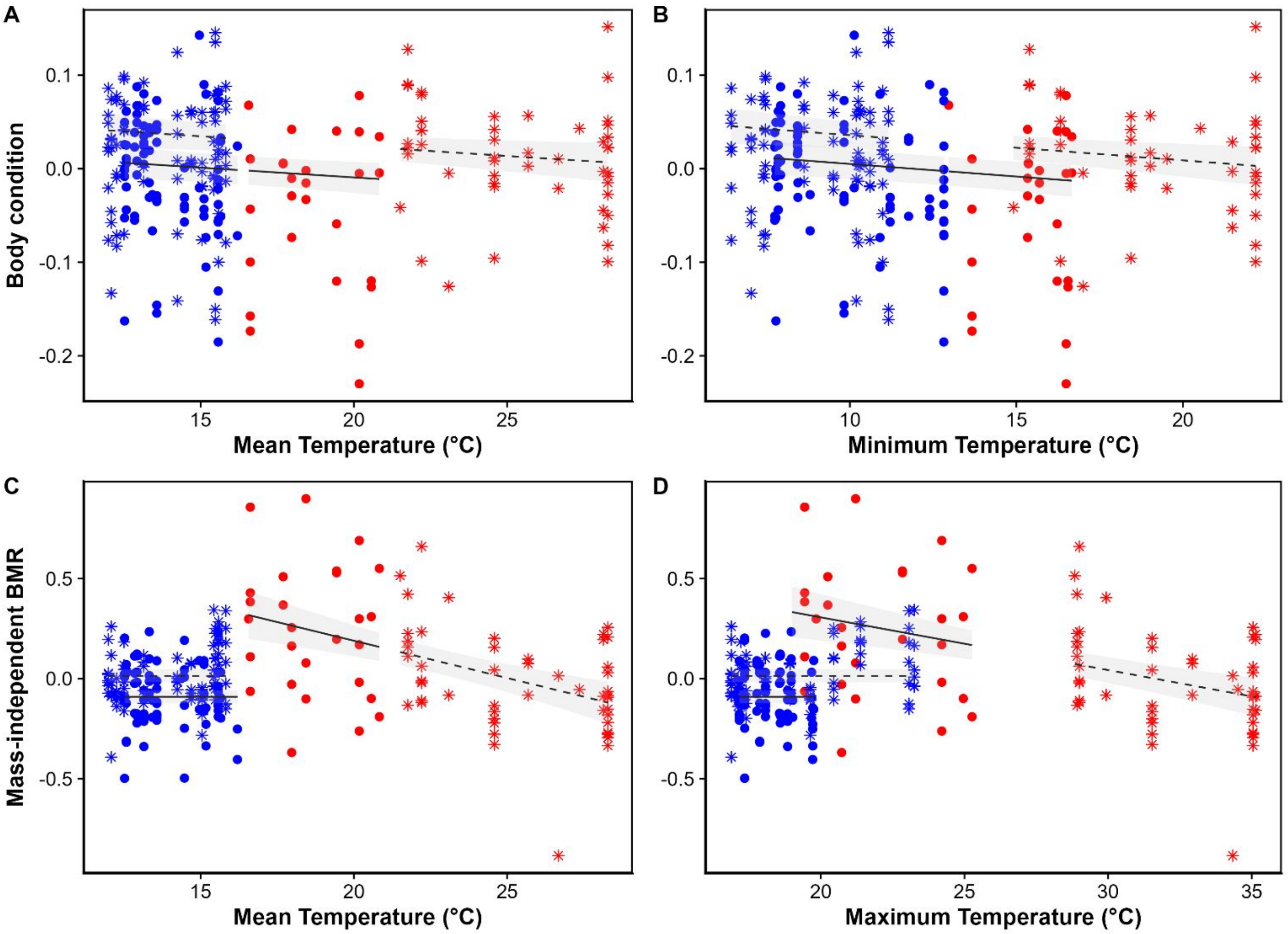

Figure S2 Body condition (A, B) and mass-independent BMR (C, D) plotted against 7-day mean, minimum, and maximum air temperatures (°C) derived from ERA5. Panels A and C depict mean temperatures; panel B depicts minimum temperature (lowest AIC score for body condition), and panel D depicts maximum temperature (lowest AIC score for mass-independent BMR). Blue = autumn, red = summer, dots and full line = northern Portugal, stars and dashed line = southern Portugal.

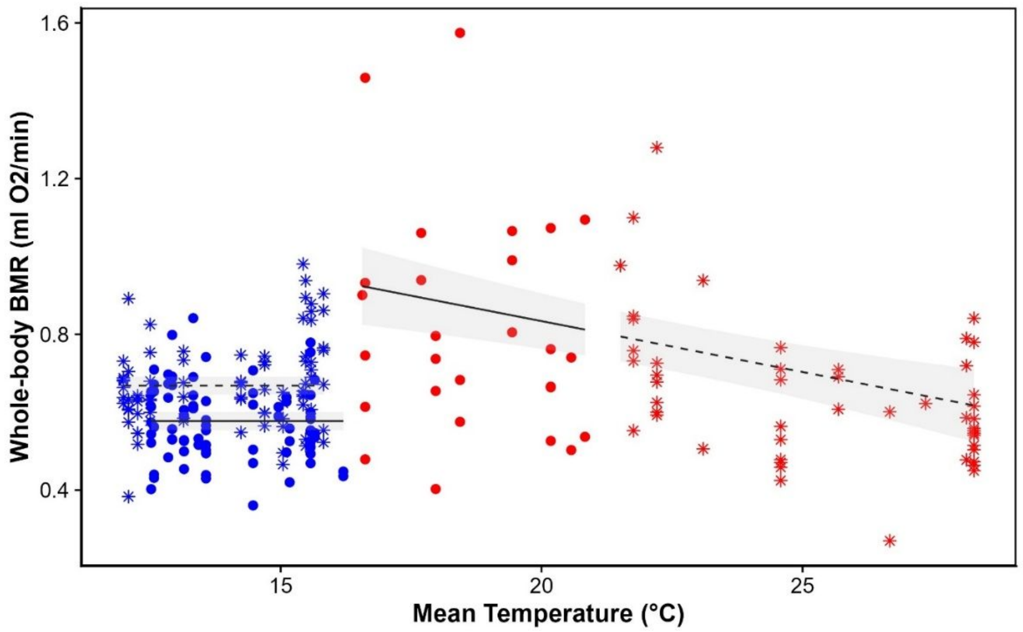

Figure S3 Whole-body BMR (ml/min) plotted against mean air temperature (°C) for the 7 days prior to BMR measurements obtained from ERA5. Blue = autumn, red = summer, dots and full line = northern Portugal, stars and dashed line = southern Portugal.
